# DIFFERENT ASPECTS OF ACCULTURATION AND COGNITIVE HEALTH AMONG MINORITY OLDER IMMIGRANTS

**DOI:** 10.1093/geroni/igz038.121

**Published:** 2019-11-08

**Authors:** Bei Wang, XinQi Dong, Melissa A Simon, Mengting Li

**Affiliations:** 1 Institute for Health, Health Care Policy, & Aging Research, Rutgers University, New Brunswick, New Jersey, United States; 2 Rutgers, The State University of New Jersey, New Brunswick, New Jersey, United States; 3 Northwestern University, Chicago, Illinois, United States

## Abstract

Immigration and acculturation process in host society have been closely linked to health consequences among the immigrant populations. However, it has been inadequately examined regarding its relationships with cognitive outcomes. Data were drawn from PINE Study. Linear regression analyses were used to examine associations between acculturation levels (media use, ethnic social relations, and language proficiency) and cognitive performance. After adjusting for potential confounding variables, higher levels of acculturation were associated with better cognitive performance. More specifically, higher levels of acculturation in aspects of media use and ethnic social relations were associated with better cognitive performance, whereas it has no significant association with the language preference aspect. Research and practice addressing health disparities and cognitive impairment should leverage efforts to provide culturally relevant resources to less acculturated populations in the US. More longitudinal studies are needed to clarify the influence of acculturation on cognitive performance and its mechanism.

